# Epidemiology of Avian Tuberculosis in Selected Districts of Oromia Region, Ethiopia

**DOI:** 10.1155/2022/6933701

**Published:** 2022-01-27

**Authors:** Tesfaye Debelu, Fufa Abunna, Gezahegne Mamo Kassa, Gobena Ameni

**Affiliations:** ^1^College of Agriculture and Natural Resources, Salale University, Fiche, Ethiopia; ^2^Collage of Veterinary Medicine and Agriculture, Addis Ababa University, Bishoftu, Ethiopia; ^3^Aklilu Lemma Institute of Pathobiology, Addis Ababa University, Addis Ababa, Ethiopia

## Abstract

Avian tuberculosis is a growing public health concern and a significant impediment to socioeconomic development, especially in developing countries, where the risk of infection is high. The disease is predominantly caused by *Mycobacterium avium* belonging to serotypes 1, 2, 3, and 6 (genotypes IS901 and IS1245) and *Mycobacterium genavense.* It mostly occurs in older birds and immunocompromised individuals due to the greater opportunity for infection with age and host immunity. A cross-sectional study anticipated to generate epidemiological information on avian tuberculosis was carried out from November 2016 to June 2017 at highland areas of Gerar Jarso, Ada'a (midland), and Boset (lowland) districts of Oromia region, Ethiopia. Two hundred seventy-three village chickens comprising local breeds, exotic breeds, and crossbreeds of both sexes were used in the study. Single intradermal avian tuberculin test, postmortem inspection of positive reactors, mycobacteriological culturing, and histopathological examination were used to assess information on the epidemiology of the disease. Subsequently, avian tuberculin test revealed an overall apparent prevalence of 11.4% (31/273) and a specific prevalence of 6.8% (6/88) in the highland, 13.4% (13/97) in the midland, and 13.6% (12/88) in the lowland study districts. Besides, it signified a higher odd of exposure in crossbreeds and females as compared to locals and males. In addition, greater odd of exposure was observed in chickens at mid- and low altitudes as compared to those at the highland. Moreover, 40.9% (9/22) of positive reactor chickens sacrificed for necropsy showed gross pathological lesions. Similarly, histopathological examination revealed a granuloma characterized by central necrosis and peripheral mononuclear lymphocytes. Nevertheless, only 0.02% (2/120) of the cultured tissues had shown colonial growth up to 12 weeks of incubation, and both were seen on sodium pyruvate-enriched Lowenstein–Jensen medium slants. Generally, the study revealed an overall increment of the apparent prevalence of avian tuberculosis with decreasing altitude. Besides, it signified a relative breed and sex variation in the risk of acquiring the disease, with crossbreeds and female chickens having higher odds of exposure.

## 1. Introduction

Avian tuberculosis is found worldwide, most commonly in small, backyard flocks and in zoo aviaries; it is rarely found in young flocks. The disease is caused by the *Mycobacterium avium* complex (MAC). *Mycobacterium avium* is very resistant and can survive in soil for ≥4 years, in 3% hydrochloric acid for ≥2 hours, and in 2% sodium hydroxide for ≥30 minutes [1].

Infected birds and contaminated water and soil are the main sources of infection since the bacteria can survive for several months in the environment [2]. The most common route of infection for susceptible birds is the alimentary tract. The respiratory tract is also suggested as a potential source of infection. The disease gets transmitted to the susceptible birds by ingestion of contaminated feed and water and inhalation of aerosolized infectious organisms. Persistence within flocks and human exposure are associated with keeping older stocks without following adequate cleanliness and hygiene [3].

Despite the paucity of information on avian tuberculosis of chickens in Ethiopia, sufficient evidence suggests that the disease occurs at significant levels in domestic cattle. For example, MAC-like organisms were isolated on culture from dairy cattle in East Shewa, Central Ethiopia [4]. The report by Tadesse et al. [5] has also revealed the highest numbers of MAC isolation in chickens from the same area, East Shewa, and a large association of chickens with other domestic animals such as cattle, sheep, and goats. More than 95% of chickens in the country are also produced under the backyard system, where there is poor hygienic standard, strong physical contact between chickens and their owners, and high risk of acquiring zoonotic diseases such as avian TB. However, studies conducted on avian tuberculosis are very scant [6]. Hence, it was crucial to generate additional epidemiological information on the apparent prevalence and associated risk factors of avian tuberculosis in the backyard chicken production system at different agroecological zones of the country, which helps to design appropriate control strategies of the disease.

## 2. Materials and Methods

### 2.1. Study Areas

The study was conducted from November 2016 to June 2017 at three purposively selected districts, namely, high areas of Gerar Jarso, Ada'a, and Boset districts located at mid- and low altitudes, respectively, of Oromia, Ethiopia.

Gerar Jarso district is located in the North Shewa zone of Oromia regional state, 114 km north of Addis Ababa at 8.54°–10.23° N and 37.56°–39.24°E. The total area of the district is 42,763 hectares, of which 52%, 41%, and 7% are the highland, midland, and lowland, respectively. The minimum altitude of the district is 1080 m, and the maximum is 3541 m above sea level. The average minimum and maximum annual rainfall are 793 mm and 1443 mm, respectively, while the average minimum temperature is 10°C and maximum temperature is 32°C. The total population of the district is 67,298 (34,462 males and 32,836 females). There are 99,000 chickens in the district of which 84,000 (84.8%) are local and 15,000 (15.2%) are exotic breeds [7].

Ada'a district is found 47 km southeast of Addis Ababa, the capital of Ethiopia. About 90% of the district belongs to the subtropical agroclimatic zone having an altitude ranging from 1500 to over 2000 m above sea level. The district receives an annual rainfall of 851 mm with the annual minimum and maximum temperature of 11 and 29°C, respectively. Though the district is most known for cereal crops (mainly teff and wheat) and legumes, livestock production is an integral part of the system. Cattle, small ruminants, poultry, and equines are the major livestock species kept with fast-growing smallholder dairy production. The district has a total livestock population of 264,310 and a total poultry population of 107,554, of which 72,541 are exotic breeds and the remaining 16,700 and 18,313 are local breeds and crossbreeds, respectively [8].

Boset is one of the districts in the Oromia Region of Ethiopia. It is part of the East Shewa zone located in the Great Rift Valley. The district is bordered on the south by the Arsi Zone, on the west by the Awash River which separates it from Adama, on the north by the Amhara Region, and on the east by Fentale. The administrative center of the district is Welenchiti. This district is predominantly level land with undulating features; almost 90% is less than 1500 meters above sea level; Boset Guddo is the highest point. A survey of the land in this district shows that 26.2% is arable or cultivable, 30% is pasture, 15.8% is forest, and the remaining 28% is considered barren, degraded, or otherwise unusable. Fruits and vegetables are important cash crops of the district. Boset has 97 kilometers of dry-weather and 103 of all-weather roads, for an average of road density of 136.8 kilometers per 1000 square kilometers. About 87% of the urban, 36% of the rural, and 45% of the total population have access to drinking water. The 2007 National Census reported a total human population of 142,112, of whom 73,925 were men and 68,187 were women; 26,514 or 18.66% of its population were urban dwellers (Wikipedia, 2014). The total chicken population of the Boset district is 107,095, of which 95,070 are local and 12,025 are exotic [9].

Three peasant associations were selected from each district based on their chicken population and accessibility to seasonal and/or all-weather roads.

### 2.2. Study Animals

The study animals were adult and young adult chickens of both sexes, local breeds, exotic breeds, and crossbreeds reared under the backyard production system. Information about the age and breeds of chickens was obtained from farmers themselves. Accordingly, pullet chickens which had begun laying were considered to be young adults; and young cocks which were in the same batch with the laying pullets were also considered in the same manner. Concerning the breed of chickens, using their local language, Afaan Oromo, owners categorized indigenous breeds as “Habesha,” exotic breeds as “Matika,” and crossbreeds as “Sanyi maka.”

### 2.3. Study Design and Sampling Methodology

A cross-sectional study design was used primarily to determine the apparent prevalence of avian tuberculosis in selected districts of Oromia region, Ethiopia. Three districts, namely, Gerar Jarso, Ada'a, and Boset, were purposively selected from high, mid-, and low altitudes of the region, respectively. Moreover, multistage sampling method was used to identify peasant associations (PAs) within the districts, households in the PAs, and study animals in the households. Thus, from each district, three PAs were selected based on their chicken population and road accessibility; and then, 10 farmers who had chickens and were willing to participate in the study were selected from different villages of each PA. Moreover, adult/young adult chickens in the households were selected using systematic random sampling for avian tuberculin test. Avian tuberculin test positive birds were sacrificed for postmortem examination, and postmortem pathological samples for cultural isolation of the etiologic agents and histopathological examinations were collected.

### 2.4. Sample Size Determination

The desired sample size (*n*) was calculated according to the formula given by Thursfield [10], considering 6.3% prevalence from the previous study undertaken in Central Ethiopia by Sultan et al. [6], 95% confidence level, and 5% precision.

(1) $$\begin{array}{c} n=1.96^{2}P_{\exp}\left(1-P_{\exp}\right),\\ d^{2}, \end{array}$$

where *n* is the required sample size, *P*_exp_ is the expected prevalence, and *d* is the desired absolute precision. Substituting values in the formula yielded the following result:

(2) $$\begin{array}{c} 1.96^{2}\times 0.063\left(1-0.063\right)=91,\\ {\left(0.05\right)}^{2}. \end{array}$$

Thus, the calculated sample size was 91 chickens. However, to increase precision and maximize the efficiency of the study, 91 chickens were considered to be the share of each district, and the total sample size was 273 chickens, which is three-fold of the calculated sample size.

### 2.5. Avian Tuberculin Test

Single intradermal avian tuberculin test was performed on selected chickens of volunteer farmers in the study districts by following the principles described by Fulton and Thoen [11]. Before injecting the avian purified protein derivatives (PPDs) which were obtained from Addis Ababa University, Aklilu Lemma Institute of Pathobiology, TB Research Laboratory, the wattle of a chicken to be injected was thoroughly inspected for any abnormality that could create confusion with the reaction of the PPD. Then, 0.1 ml of 2500 IU/ml avian PPD was injected intradermally on the right wattle of selected chickens using a sterile insulin syringe of 1 ml capacity, and the sites of injection were marked with a permanent marker. Moreover, in circumstances when the number of chickens in the flock was more than ten, a cord was tied on one of the legs of tested chickens to differentiate them from the remaining flock.

The test result was appreciated for the presence of allergic reactions 48 hours after the injection of the avian PPD. Based on the principle described by Dhama et al. and Thursfield [2, 10], a positive reaction was identified as a hot and edematous swelling at the site of inoculation or by the presence of a small firm nodule of approximately 5 mm or above in diameter which was clearly observed by comparing with the left wattle which was used as a control. In test negative chickens which had a tie on their legs, the cords were removed and left only on test positive chickens in the flock which later were purchased and sacrificed for postmortem examination.

### 2.6. Postmortem Examination

Avian tuberculin test positive chickens were purchased from farmers and transported to the College of Veterinary Medicine and Agriculture, Addis Ababa University postmortem room, and humanely sacrificed for postmortem examinations to look for lesions related to avian tuberculosis. After the chickens were made unconscious by dislocating their neck, feather covering around the entire ventral part of the chickens was removed to avoid the risk of contamination of internal organs with external contaminants on chickens' feather. Subsequently, incision was made from the beak to the anus following the ventral midline of the chickens, and the underlying soft and boney tissues were dissected with sterile scissors and bone shears to expose the internal organs for postmortem examination. This was done in accordance with the procedures of Zander and Mallison [12].

All internal organs at necropsy with special attention to the liver, spleen, and intestine were examined, and any observed gross lesions were recorded. Subsequently, the gross lesions were characterized, and their distributions were described. A pair of any suspected organs and tissues such as the liver, spleen, and intestine of all slaughtered chickens with visible or invisible gross pathological lesions was sliced and sampled using sterile surgical blades to minimize the possibility of missing obscure tubercular lesions. To avoid the risk of cross-contamination and false culture positivity of sampled tissues and organs, a separate sterile surgical blade was used for each and every organ and tissue sampled. Meanwhile, the tissue samples were kept in universal bottles filled with approximately 5 ml of phosphate-buffered saline solution and 10% buffered formalin for mycobacteriological culturing and histopathological examinations, respectively. Then, the tissue samples were transported using an ice box with ice packs to the College of Veterinary Medicine and Agriculture (CVMA), Addis Ababa University bacteriology laboratory. Tissue samples that were collected for mycobacteriological culturing were stored at −20°C until transported to the Aklilu Lemma Institute of Pathobiology (ALIPB) TB laboratory for cultural isolation and characterization of MAC and other suspected *Mycobacterium* species, while tissue samples preserved in 10% buffered formalin were kept at room temperature until transported to the National Animal Health Diagnostic and Investigation Center (NADIC) for histopathological examination.

### 2.7. Histopathological Examination

Tissues collected for this purpose from organs showing pathological gross lesions and samples of the liver, spleen, and intestine suspected for obscure lesions and preserved in 10% buffered formalin were transported to the National Animal Health Diagnostic and Investigation Center at Sebeta, Oromia, Ethiopia. The tissues were dehydrated in different grades of ethanol (70%, 95%, and 100%), cleared in xylene, and refixed with formalin in an automatic tissue processing machine for about 20 hours and 30 minutes. Then, the tissues were embedded in paraffin using an embedding machine and cut into thin sections of 4 to 5 *μ*m using a microtome following the procedures described by Zander and Mallison [12]. Subsequently, the tissue sections were stained with haematoxylin and eosin and prepared for microscopic examination [13].

### 2.8. Culturing

Tissue samples were transported in a cold chain using an ice box packed with ice blocks to the Aklilu Lemma Institute of Pathobiology (ALIPB) TB laboratory, Addis Ababa, Ethiopia. Following the protocols of OIE [13] for isolation of mycobacteria from tissues, the samples were sectioned into pieces using sterile scissors and homogenized by pestle and mortar. The homogenate was dispensed into the falcon tubes, decontaminated by adding an equal volume of 4% NaOH, and centrifuged at 3000 rpm for 15 minutes. After centrifugation, the supernatant was discarded, whereas the sediment was neutralized by 10% HCl using a drop of phenol red as an indicator. Neutralization was achieved when the color of the solution changed from purple to yellow. Then, 0.1 ml of suspension was spread onto a slope of the Lowenstein–Jensen (LJ) medium. For each sample, duplicates of the LJ medium, one enriched with sodium pyruvate and the other enriched with glycerol, were used to increase the chance of growth of both *Mycobacterium tuberculosis* and nontuberculosis mycobacterium species (NTM) such as *Mycobacterium* species under the *Mycobacterium avium* complex (MAC). Cultures were incubated at 37°C in a slant position for one week and in the upright position for 12 weeks with weekly observation for mycobacterial growth, dehydration, and overhydration of the culture.

### 2.9. Acid-Fast Staining

Ziehl–Neelsen's acid-fast staining was performed on two positive cultures to confirm the presence of acid-fast bacilli following the protocols described by Quinn et al. and the WHO [14, 15].

### 2.10. Data Analysis

Descriptive statistics, Fisher's exact test, and univariable logistic regression were performed to analyze the data using Stata version 13.0 software packages and Statistical Package for Social Sciences (SPSS) version 20.0. For all analyses that were performed, 95% CI and *p* value <0.05 were set for statistical significance of an estimate.

## 3. Results

### 3.1. Epidemiological Investigation of Avian Tuberculosis

**Figure 1:**
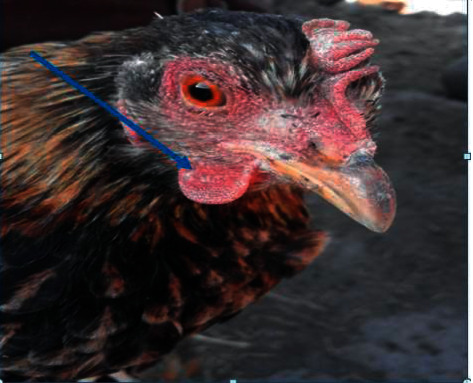
Avian intradermal tuberculin test positive chickens with edematous swelling on the right wattle at the site of inoculation of the avian PPD observed in this study (indicated by an arrow).

**Figure 2:**
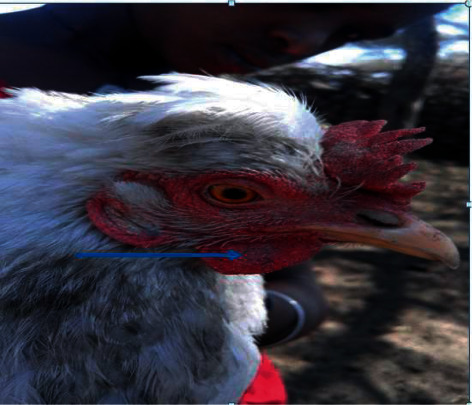
Firm erythematic nodular swelling on the right wattle of the avian intradermal tuberculin test positive chicken (indicated by an arrow).

A total of 273 chickens from Gerar Jarso, Ada'a, and Boset study districts were inoculated with avian PPDs at their right wattle to investigate the epidemiology of avian tuberculosis in the districts. Among them, 31 chickens, 6 from Gerar Jarso (highland), 13 from Ada'a (midland), and 12 from Boset (lowland), were identified as positive reactors to the avian tuberculin test (Table 1 and Figures 1 and 2).

To evaluate the spatial distribution of the disease, a total of nine peasant associations from the three study districts, 3 from Gerar Jarso, 3 from Ada'a, and 3 from Boset, were included in the present study. Accordingly, one or more chickens were identified as avian tuberculin test positive in eight of them, except one peasant association in Gerar Jarso district, where no single positive reactor chicken for avian tuberculin test was recorded. Hence, the current study revealed that avian tuberculosis is well prevalent in the selected peasant associations in the study districts.

The effect of host-related and environmental risk factors such as breed, sex, and altitude as predisposing factors for avian tuberculosis in chickens was also evaluated using the univariable logistic regression model. In view of this, the comparison between breeds as the risk factor for the occurrence of the disease revealed a twofold higher odd of exposure of crossbreed chickens (OR = 2.36; 95% CI: 0.44, 12.75) as compared to local breed chickens involved in the present study. However, this difference was not statistically significant (*p* > 0.05) (Table 2).

In addition, within a breed, the statistical estimation of the effect of sex as the predisposing factor of chickens to avian tuberculosis (ATB) showed the odd of exposure of female chickens (OR = 2.87; 95% CI: 0.66, 12.5) to be nearly three times the odd of exposure of males. Nevertheless, the difference was not statistically significant (*p* > 0.05) (Table 2).

Likewise, the study also assessed the effect of altitude as the predisposing factor for avian TB in chickens. Consequently, the result connoted a twofold increment in the odds of exposure of chickens in the midaltitude (OR = 2.12; 95% CI: 0.77, 5.83) and low altitude (OR = 2.16; 95% CI: 0.77, 6.04) to the disease as compared to chickens in the high altitude of the districts selected for this particular study, though the difference was not statistically significant (*p* > 0.05) (Table 2).

### 3.2. Necropsy Findings

Postmortem examination was carried out on avian tuberculin test positive chickens. From the total of 31 positive reactor chickens, 22 chickens were sacrificed for necropsy. Nevertheless, the remaining 9 positive reactor chickens were not obtained for necropsy due to reasons such as reluctance of owners to sell broody chickens, death, and disposal of test positive chickens and because of other acute and subacute diseases such as Newcastle disease before handing over them for necropsy.

Among the sacrificed chickens, 40.9% (9/22) of them showed postmortem gross lesions such as organomegaly, organ atrophy, miliary nodules, and diffused or petechial hemorrhages on the liver and/or spleen and intestine (Table 3).

**Figure 3:**
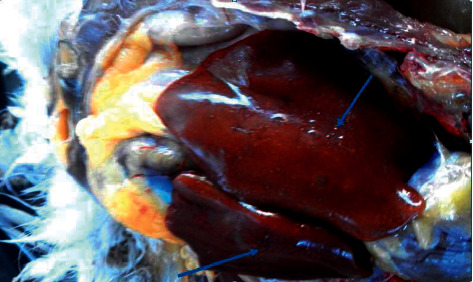
Arrows showing grayish-yellow to grayish-white nodular swelling on the liver tissue of the avian intradermal tuberculin test positive chicken observed upon postmortem examination.

The miliary nodular lesions observed on a liver of a local breed female chicken in the present study were grayish-yellow to grayish-white, pin-point to irregularly round, and few to numerous on different parts of the organ, approximately measuring up to 2 cm in diameter and raised above the surface of the affected organ (Figure 3). Nevertheless, no calcification or caseation was visualized in the nodules.

**Figure 4:**
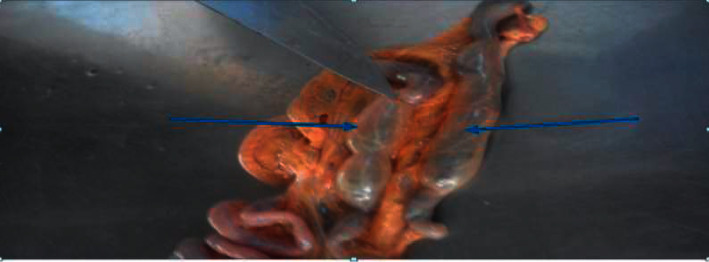
Arrows showing paired ceca of the avian intradermal tuberculin test positive chicken filled with air and cecal walls devoid of epithelial cells and connective tissue as observed upon postmortem examination.

Moreover, one chicken showed splenomegaly, and an exotic female chicken from Gerar Jarso showed a pale and highly atrophied liver and paired ceca filled with air. Besides, the atrophied part of the cecal wall of the intestine in this chicken was devoid of epithelial cells and connective tissue, except a thin fascia which accounts for most of the cecal part of the intestine (Figure 4). However, upon antemortem examination, this exotic chicken had a very good body condition and shiny feathers with good physical appearance.

**Figure 5:**
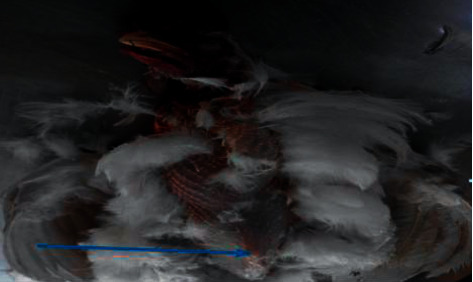
Arrow showing the prominent keel bone and wasting of the breast muscle observed on the avian intradermal tuberculin test positive chicken upon postmortem examination.

The other striking feature that was visualized during postmortem examination of a well-matured positive reactor male chicken with good body condition and feather quality was remarkable wasting of the breast muscle, a skeletal muscle attached to the keel bone, leathery appearance of the skin covering the breast muscle, and a prominent keel bone having a knife-edged appearance (Figure 5).

### 3.3. Mycobacteriological Culture Results

A total of 60 tissue samples collected from different organs including the liver, spleen, intestine, lungs, and heart were cultured on a pair of Lowenstein–Jensen (LJ) media (prepared from the standard LJ base medium having optimum pH and supplemented with 12 ml glycerol and 1000 ml egg suspension using a standard protocol). A pair of LJ slants, one enriched with sodium pyruvate and the other enriched with glycerol, were used, and hence, a total of 120 slants were prepared (all the 60 tissue samples were seeded on sodium pyruvate-enriched and glycerol-enriched LJ slants). Of these, only 0.02% (2/120) of them had shown colonial growth up to 12 weeks of incubation, and both were seen on sodium pyruvate-enriched LJ slants. However, no more than a single slight colony was appreciated on both LJ slants; and hence, after smearing for Ziehl–Nielsen staining, it was not sufficient for molecular typing of the isolates.

### 3.4. Acid-Fast Staining

Ziehl–Nielsen staining technique performed on the colonies from the two culture-positive LJ slants revealed that the organisms were acid-fast rods suggesting that they are under the genus *Mycobacterium*.

### 3.5. Histopathological Findings

**Figure 6:**
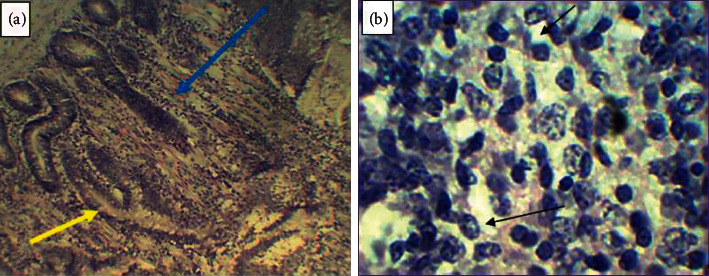
Tubercular granuloma on intestinal tissue (a) characterized by central necrosis indicated by the blue arrow and peripheral inflammatory cells shown by the yellow arrow and lymphoid depletion on the spleen (b) indicated by the black arrows.

Histopathological examination was carried out on samples collected from gross pathological lesions. From a total of ten histopathological slides prepared from strongly suggestive gross pathological lesions on the liver, spleen, and intestine of seven positive reactor chickens, 40% (4/10) of them revealed tubercular granuloma characterized by central necrosis and peripheral inflammatory cells, which were mainly mononuclear lymphocytes and macrophages. Moreover, lymphoid depletion of the cortical part of the spleen with empty cystic structure at the sites of lymphoid depletion, a characteristic feature of diseases caused by *Mycobacterium* species, was also visualized (Figure 6). These histopathological findings were obtained from postmortem tissues of three different positive reactor female chickens. Furthermore, out of the four postmortem tissues which had shown tubercular granuloma, 50% (2) of them were intestines, and the remaining two were the liver and spleen.

## 4. Discussion

The present study attempted to investigate the epidemiology of avian tuberculosis in selected districts of Oromia region, Ethiopia. It revealed that the overall apparent prevalence of avian tuberculosis in scavenging domestic chickens in the area was 11.4%. It also connoted the specific prevalence of the disease in the three study districts as 6.8% in Gerar Jarso (highland), 13.4% in Ada'a (midland), and 13.6% in Boset (lowland), implying that the prevalence of the disease decreases with increasing altitude [16]. The study is also the first of its kind to explore the prevalence of avian tuberculosis in the central highland of Ethiopia. Nevertheless, these figures seem a little bit bigger as compared to a similar study conducted in Central Ethiopia which reported a 6.3% prevalence of the disease from Adama town [5]. This discrepancy could be due to the temporospatial disparity between the two studies, which implies that the prevalence of avian tuberculosis in Central Ethiopia and hence its public health implications in traditional chicken producers of the area could differ over time and location. Another study conducted on domestic chickens at Shashamene district also reported the prevalence of avian tuberculosis in the district as low as 4.23% [6]. This variation could also be related to the variation in the spatial distribution of the disease and other host-related factors in the areas that negatively affected the prevalence of the disease.

Recently, a study conducted on domestic chickens in selected sites of Ethiopia also revealed the prevalence of avian tuberculosis in semi-intensive exotic chickens in Bishoftu town as 5.85% [17]. This result also varies from the results of this study probably due to the difference between the management systems under which the chickens were kept because in the semi-intensive production system, the farm hygiene and other husbandry practices are better than the backyard production system. However, in the backyard production system, the awareness of farmers regarding the hygienic and other management systems is purely traditional. Hence, these hygienic and management problems could strongly hamper the health of the public [18] and productivity of the traditional chicken production system in our country and favor the prolonged existence and transmission of the environmentally resistant bacteria, MAC, which are the etiologic agents of the disease in chickens, and the risk of zoonosis for humans as well. According to Dhama et al. [2], wild birds such as sparrows, crows, and pigeons may be infected with *M. avium* and may spread it to poultry flocks. Consequently, since scavenging chickens are in unlimited interaction with wild birds, they could easily transmit the disease to the scavenging domestic chickens. Moreover, Sultan et al. [6] also reported that most owners give irregular grain supplements to their chickens which could attract the wild birds and increase the risk of contact between wild birds and domestic scavenging chickens. Hence, there is a strong likelihood that scavenging chickens may acquire the infection from the soil in a contaminated environment with the droppings of chronic carrier chickens and wild birds [19]. Thus, it is logical to appreciate the increased prevalence of avian tuberculosis in scavenging chickens as compared to semi-intensively managed ones.

A univariable statistical evaluation of the major host-related and environmental risk factors of avian tuberculosis in chickens also revealed breed as one of the major host-related risk factors of the disease. Accordingly, though the disease was well prevalent in all breeds of the traditionally produced domestic chickens in the study districts, it revealed higher odds of exposure of crossbreed chickens (OR = 2.36; 95% CI: 0.44, 12.75) as compared to the odd of exposure of local breeds involved in the study, but the difference was not statistically significant (*p* > 0.05). This finding was also in agreement with the finding of a study conducted on semi-intensive poultry farms of Bishoftu town and village chickens at Bahir Dar and its surrounding by Kindu and Getaneh [17] who reported a significantly (*p* < 0.05) higher rate of avian tuberculin positivity of exotic breed chickens as compared to local breeds. This implies that breed is a putative risk factor which predominantly predisposes chickens to avian tuberculosis. Thus, exotic breed and crossbreed chickens have shown a higher risk of exposure to the disease as compared to the local breed.

On the contrary, a univariable logistic regression result has shown a significantly (*p* < 0.05) reduced odd of exposure of exotic breed chickens as compared to the local ones. Such a controversial result to the previously documented scientific information concerning the innate resistance of local and exotic breeds could be due to the disproportionate number of local and exotic breeds involved in the study.

Evaluation of the sex of chickens as the host-related trait indicated that 29 of the 31 chickens (93.6%) that were positive for avian tuberculin test were females. Hence, the univariable logistic regression model revealed a nearly threefold higher odd of exposure of female chickens to avian TB as compared to their male counterparts (OR = 2.87; 95% CI: 0.66, 12.5). Nevertheless, the difference was not statistically significant (*p* > 0.05). This result also agrees with similar studies in domestic chickens which were conducted at Adama, Sebeta, and Debre Berhan towns of Central Ethiopia by Tadesse et al. [5] and at Shashamene district of the West Arsi zone of Oromia by Sultan et al. [6], who reported sex variation on the occurrence of avian TB with slightly higher occurrence in female adult chickens than male sex counterparts. This might be due to the fact that farmers keep female chickens for a prolonged period of time for egg production as compared to males, and this longer production time could increase the risk of exposure of female chickens to the etiology of the disease, MAC, which is highly resistant to environmental extremes, and can survive in the environment for a prolonged period of time, usually for ≥4 years [1]. Moreover, the number of male chickens (*N* = 42) included in the study was also by far less than the number of female chickens (*N* = 231) because most farmers usually keep only 1 male chicken in their breeding females or others might not keep them at all. Hence, this disproportionate involvement of male and female chickens in the intradermal avian tuberculin test in this particular study could also cause deviation of the true effect of sex as the risk factor of avian tuberculosis.

The study also revealed the effect of altitude as a risk factor for the occurrence of avian TB in chickens. Accordingly, from the three agroecological zones compared in this study, the odds of exposure of chickens at midland (OR = 2.12; 95% CI: 0.77, 5.83) and lowland (OR = 2.16; 95% CI: 0.77, 6.04) altitudes to avian TB were observed to be increased by twofold as compared to the odd of exposure of chickens at high altitude, but this difference was not statistically significant (*p* > 0.05). The result was also in agreement with similar studies on domestic chickens conducted at Bishoftu town and Bahir Dar and its surrounding by Kindu and Getaneh [17] and at Adama, Sebeta, and Debre Berhan towns of Central Ethiopia by Tadesse et al. [5] who reported the contribution of mid- and low altitudes as a risk factor for the occurrence of avian tuberculosis in chickens to be higher as compared to high altitude from where they had identified no positive reactor chicken to the avian intradermal tuberculin test conducted in their respective studies. This implies that, even though MAC is known to resist and survive in all environmental extremes except direct sun light, the hot and less humid environment of the mid- and low altitudes might better favor the growth and survival of the organisms, which are the major etiology of avian tuberculosis in chickens, and hence increase the risk of exposure of chickens in the area as compared to high altitudes where the environment is colder and humid.

According to some scholars, postmortem examination of gross lesions has been commonly used for diagnosis of avian TB concurrently with tuberculin test [20]. In this study, the postmortem detection rate of tuberculous lesion from the tuberculin test positive chickens was 40.9% (9/22). However, gross lesions were not detected on the remaining 59.1% (13/22) of chickens, and this might be associated with the possibility of missing the lesions of recent infection and existence of obscure lesions which are common in mycobacterial infections [21]. However, due to the lack of facility and sufficient fund for the study, molecular detection of the organism from postmortem samples was not undertaken.

Necropsy finding in the present study also evidenced gross pathological lesions which were suggestive for avian tuberculosis such as knife-edged appearance of the keel bone and sloughing and disappearance of the wall of the cecal part of the intestine, except a thin fascia which was filled with air, in a chicken with a very good physical appearance and shiny feathers upon antemortem inspection. This implies that a chicken which is performing well and having good physical appearance could be positive for avian tuberculosis and be a risk factor for the health of consumers [18]. According to Dhama et al. [21], in most cases, an infected bird without overt clinical signs may serve as a carrier, which results in the persistence of infection in flocks. Hence, this result is also in agreement with the report of this scholar.

Nevertheless, the culture of postmortem tissue samples with gross or suspected avian TB lesions collected from the liver, spleen, intestine, and lungs of reactor chickens to avian tuberculin test was hardly responded. From a total of 60 tissue samples cultured in pairs on 60 pyruvate-enriched and 60 glycerol-enriched Lowenstein–Jensen medium slants, which total 120, only 0.02% (2/120) had shown culture positivity up to 12 weeks of incubation at 37°C. This could be either due to poor specificity of the test to rule-in the true positive chickens to avian tuberculosis or due to problems with the efficiency of the media used for culturing, Lowenstein–Jensen medium, to support MAC or the prolonged time it requires for the growth of these organisms. Studies conducted in cattle populations free of bovine TB have shown the specificity of the single intradermal comparative tuberculin test to be between 78.8% and 100% with a median of 99.5% [22]. In a more recent study using latent class analysis without a gold standard, the sensitivity and specificity of the test were reported to be 52.9–60.6% and 99.2–99.8%, respectively [23].

It could also be due to the problem with the temperature at which the culture was incubated, which is lower than the average body temperature of domestic chickens (41°C). This finding is also in agreement with the finding of Kindu and Getaneh [17] who reported that, from the total of 34 samples taken from different organs of 12 slaughtered tuberculin reactor chickens and cultured on LJ slants, 17 were inoculated on sodium pyruvate-enriched and the other 17 on glycerol-enriched LJ slants, none of them have shown colonial growth up to 8 weeks of incubation. They also suggested the reason for the failure of MAC growth as inappropriateness of the incubation temperature (37°C) since it does not fit the natural hosts' internal body temperature (41°C).

According to Thoen [24], who compared the efficiency of four culture media for the isolation of the *Mycobacterium avium* complex from 197 porcine tissues with or without microscopic granulomas and acid-fast bacilli, a significantly greater number of isolates (*p* < 0.05) were obtained on the Middlebrook 7H10 medium with sodium pyruvate than on the Stonebrink medium, Herrold egg yolk agar medium, or Lowenstein–Jensen medium. The time required to grow *M. avium* complex on the Lowenstein–Jensen medium was also significantly greater than the time required to observe growth on the Stonebrink, Middlebrook 7H10, or Herrold egg yolk agar medium (*p* < 0.05) [24]. This implies that the Lowenstein–Jensen medium is not the medium of choice for culturing MAC to obtain a better number of isolates within the optimum period of time. On the contrary, Tadesse et al. [5] reported a culture positivity of 6.3% (6 out of 95) chickens' postmortem tissue samples cultured on pyruvate-enriched Lowenstein–Jensen slants. Similarly, Sultan et al. [6] also reported a culture positivity of 50% (3 from 6) chickens with gross lesions on the same medium, Lowenstein–Jensen medium. Hence, these controversial results signify the issue of MAC culturing to be an area for future research.

Histopathological examination of postmortem tissues collected from similar organs (liver, spleen, and intestine) from which tissues for culturing were collected, on the contrary, revealed a tubercular granuloma characterized by central necrosis and peripheral inflammatory cells, mainly of mononuclear lymphocytes and macrophages. Moreover, lymphoid depletion of the cortical part of the spleen with empty cystic structure at the sites of lymphoid depletion, which is also a characteristic feature of avian TB, was also visualized on the histopathological slides. This finding agrees with the findings of Tadesse et al. [5] who reported the presence of granuloma characterized by caseonecrotic cores that were surrounded by a broad ring of palisading epithelioid cells, macrophages, and multinucleate giant cells with a moderate mixture of heterophils, lymphocytes, and plasma cells. Hence, the result supported and confirmed the tuberculin test result and necropsy findings, but it contradicted the poor cultural response observed in this study, suggesting that poor culture positivity may be due to problems related to the nature of the bacteria and/or poor efficiency of the culture media, LJ medium, to support *Mycobacterium* species under the *Mycobacterium avium* complex and the prolonged time it requires for the growth of MAC on the LJ medium.

## 5. Conclusions

The current epidemiological investigation in village chickens conducted in Central Ethiopia using avian tuberculin test revealed an overall increment of the apparent prevalence of avian tuberculosis and its decrease with increasing altitude. Besides, it signified a relative breed and sex variation in the risk of acquiring the disease, with crossbreeds and female chickens having higher odds of exposure. An attempt made to isolate and characterize the species of mycobacteria from postmortem samples of positive reactor chickens during this particular study was hardly successful. However, due to the lack of facility and sufficient fund for the study, molecular detection and confirmation of the organism from postmortem samples were not undertaken.

## Figures and Tables

**Table 1:** Summary of the spatial distribution of chickens tested with avian tuberculin test in the present study and prevalence of avian TB with sex, breed, and altitude.

Variables	Categories	Percentage of chickens tested with the avian PPD, % (*N*)	Percent positive, % (*N*)	Prevalence of avian TB, % (*N*)	95% confidence interval

Sex	Male	15.4 (42)	6.5 (2)	4.8 (2/42)	[0.6, 16]
	Female	84.6 (231)	93.5 (29)	12.6 (29/231)	[9,18]

Breed	Local	70.7 (193)	90.3 (28)	14.5 (28/193)	[10, 20]
	Exotic	26.7 (73)	3.2 (1)	1.4 (1/73)	[0.03, 7]
	Cross	2.6 (7)	6.5 (2)	28.6 (2/7)	[4, 71]

Altitude	Highland	32.2 (88)	19.4 (6)	6.8 (6/88)	[3, 14]
	Midland	35.6 (97)	41.9 (13)	13.4 (13/97)	[7, 22]
	Lowland	32.2 (88)	38.7 (12)	13.6 (12/88)	[7, 23]

Total		100 (273)	100 (31)	11.4 (31/273)	[8, 16]

**Table 2:** Univariable logistic regression output of host-related and environmental risk factors and their odds of exposure of chickens to avian tuberculosis.

Variables	Categories	Percent of chickens tested, % (*N*)	Percent positive, % (*N*)	Odds ratio	*p* value	95% confidence interval

Breed	Local^*∗*^	70.7 (193)	14.5 (28)	1		
	Exotic	26.7 (73)	1.4 (1)	0.08	0.013	[0.01, 0.61]
	Cross	2.6 (7)	28.6 (2)	2.36	0.319	[0.44, 12.75]

Sex	Male^*∗*^	15.4 (42)	6.5 (2)	1		
	Female	84.6 (231)	93.5 (29)	2.87	0.160	[0.66, 12.5]

Altitude	Highland^*∗*^	32.2 (88)	19.4 (6)	1		
	Midland	35.6 (97)	41.9 (13)	2.12	0.148	[0.77, 5.83]
	Lowland	32.2 (88)	38.7 (12)	2.16	0.143	[0.77, 6.04]

**Table 3:** Postmortem gross pathological lesions in positive chickens with their breed, sex, and origin.

Variables	Category	Percentage of positive reactor chickens to the avian PPD, % (*N*)	Percentage of reactor chickens positive for postmortem gross lesions, % (*N*)

Sex	Male	6.5 (2)	11.1 (1)
	Female	93.5 (29)	88.9 (8)

Breed	Local	90.3 (28)	77.8 (7)
	Exotic	3.2 (1)	11.1 (1)
	Cross	6.5 (2)	11.1 (1)

Altitude	Highland	19.4 (6)	22.2 (2)
	Midland	41.9 (13)	33.3 (3)
	Lowland	38.7 (12)	44.4 (4)

## Data Availability

The data used to support the findings of this study are available from the corresponding author. Upon logical request, they can be accessible.

## References

[1] Thoen C. (2013). *Last Full Review/revision*.

[2] Dhama K., Mahendran M., Tomar S. (2008). Pathogens transmitted by migratory birds: threat perceptions to poultry health and production. *International Journal of Poultry Science*.

[3] Dvorska L., Matlova L., Ayele W. Y. (2007). Avian tuberculosis in naturally infected captive water birds of the Ardeideae and Threskiornithidae families studied by serotyping, IS901 RFLP typing, and virulence for poultry. *Veterinary Microbiology*.

[4] Tadelle K. (1998). *Epidemiology and Zoonotic Importance of Bovine Tuberculosis in Selected Sites of Eastern Shoa, Ethiopia*.

[5] Tadesse S., Woldemeskel M., Molla B. (2004). Avian mycobacteriosis in domestic chickens from selected agro-climatic regions in Ethiopia. *International Journal of Applied Research in Veterinary Medicine*.

[6] Sultan A., Gezahegne M., Adane W., Gobena A. (2015). Preliminary study on avian tuberculosis and associated risks in domestic chickens at Shashemene district, Ethiopia. *International Peer-reviewed Journal*.

[7] Gerar Jarso District Office of Livestock Development and Fisheries (2016). *Agro-ecological Information, Livestock and Poultry Population of Gerar Jarso District, North Shewa, Oromia, Ethiopia, *.

[8] Ada’a District Office of Livestock Development and Fisheries (2017). *Agro-ecological Information, Livestock and Poultry Population of Ada Chukala District, East Shewa, Oromia, Ethiopia *.

[9] Boset District Office of Livestock Development and Fisheries (2017). *Agro-ecological Information, Livestock and Poultry Population of Boset District, East Shewa, Oromia; Ethiopia *.

[10] Thursfield M. (1995). *Veterinary Epidemiology*.

[11] Fulton R., Thoen C., Saif Y. M., Barnes H. J., Glisson J., Fadly F., Mc Dougald L., Swayne D. (2003). Tuberculosis. *Diseases of Poultry*.

[12] Collett S. R., Smith  J. A., Boulianne M. (1991). Principles of disease prevention and control. *Diseases of Poultry*.

[13] Bancroft J., Cook H. (1994). *Manual of Histological Techniques and Their Diagnostic Application*.

[14] Quinn J., Carter E., Marker B., Carter P. (1994). Mycobacterium species. *Clinical Veterinary Microbiology*.

[15] WHO (1998). *Laboratory Services in Tuberculosis Control*.

[16] Tesfaye D. (2017). *Epidemiology and Public Health Implications of Avian Tuberculosis in Selected Districts of Oromia, Ethiopia*.

[17] Kindu A., Getaneh G. (2016). Prevalence of avian tuberculosis in domestic chickens in selected sites of Ethiopia. *Journal of Veterinary Science & Technology*.

[18] Tesfaye D., Fufa A., Gezahegne M. K. (2021). A preliminary study on public health implications of avian tuberculosis in selected districts of the Oromia region, Ethiopia. *Veterinary Medicine International*.

[19] Tell L. A., Woods L., Cromie R. L. (2001). Mycobacteriosis in birds. *Revue Scientifique et Technique de l’OIE*.

[20] Gallagher J., Monies R., Gavier-Widen M., Rule B. (1998). Role of infected, non-diseased badgers in the pathogenesis of tuberculosis in the badger. *The Veterinary Record*.

[21] Dhama K., Mahendran M., Tiwari R. (2011). Tuberculosis in birds: insights into theMycobacterium aviumInfections. *Veterinary Medicine International*.

[22] de la Rua-Domenech R., Goodchild A. T., Vordermeier H. M., Hewinson R. G., Christiansen K. H., Clifton-Hadley R. S. (2006). Ante mortem diagnosis of tuberculosis in cattle: a review of the tuberculin tests, *γ*-interferon assay and other ancillary diagnostic techniques. *Research in Veterinary Science*.

[23] Clegg T. A., Duignan A., Whelan C. (2011). Using latent class analysis to estimate the test characteristics of the gamma-interferon test, the single intradermal comparative tuberculin test and a multiplex immunoassay under Irish conditions. *Veterinary Microbiology*.

[24] Thoen C. (1978). *Comparison of Four Culture Media for Isolation ofMycobacterium Avium Complex from Porcine Tissues, Diagnostic Bacteriology Laboratory, and Pathology, Toxicology, and Parasitology Laboratory, NationalVeterinary Services Laboratories, Animal and Plant Health Inspection Service*.

